# Impacts of artificial intelligence on healthcare business models and outcomes

**DOI:** 10.1108/JHOM-06-2025-0356

**Published:** 2026-01-27

**Authors:** Mus'ab Muhammad Kakale, Nicola Pinelli

**Affiliations:** Department of Management and Law, School of Economics, Università Degli Studi di Roma Tor Vergata, Roma, Italy

**Keywords:** Innovation, Technology, Artificial intelligence, Transformation, Business models, Healthcare

## Abstract

**Purpose:**

This study aims to identify what the interaction between artificial intelligence (AI) and business models (BMs) can have on healthcare (HC) outcomes and experience and also what the ethical considerations are for AI implementation in HC. The study proposes an exploratory method using data collected from HC professionals. It aims to fill the identified gaps and contribute to theory and practice.

**Design/Methodology/Approach:**

This study used an exploratory methodology based on open-ended essay questions designed on prolific.co and collected data from 29 HC professionals. These include experts in the use of AI in HC, and a content analysis was used to analyze the data.

**Findings:**

The results showed that using AI in HC can lead to the standardization of HC services, leading to improved access to quality care with reduced cost. It revealed that AI can increase HC efficiency by automating services and increasing innovations and discoveries. But there are some ethical concerns hindering the adoption of AI HC, including data privacy, bias in algorithms and liability issues.

**Research limitations/implications:**

The sample size and the lack of including the opinions of patients are the major limitations of this study. Future studies are encouraged to improve in this direction.

**Practical implications:**

This study will promote and facilitate the wider acceptance and implementation of AI in HC. It will also enable policy and decision-makers to address ethical issues hindering the adoption of AI in HC.

**Originality/Value:**

This study has filled an important research gap by revealing how AI is impacting HC BMs and outcomes. The study will facilitate the adoption of AI in HC.

## Introduction

There has been growing interest in digital technologies by practitioners and academia over the past decades, due to the rapid changes they are making to businesses and society (Evers *et al*., 2023; Katz and Koutroumpis, 2013; Martínez-Caro *et al*., 2020; Nambisan *et al*., 2017; Zott and Amit, 2010). They can disrupt current organizational activities and processes (Hacklin *et al*., 2018; Palmié *et al*., 2020), changing the overall dynamics of markets (Gomez *et al*., 2016; Khan *et al*., 2023; Kraus *et al*., 2023; Porter and Heppelmann, 2014), leading to the creation of new business models (BMs) (Attah-Boakye *et al*., 2023; Climent and Haftor, 2021; Visnjic *et al*., 2016) and influencing innovation and decision processes (Berman, 2012; Bouncken *et al*., 2021). Many firms are adopting them to exploit business opportunities and acquire benefits (Berman, 2012; Krakowski *et al*., 2023; Tamvada *et al*., 2022). Notwithstanding, healthcare (HC) is experiencing a lack of personnel (Michel and Ecarnot, 2020), inefficiency (OECD, 2017) and inequalities (Hamed *et al*., 2020), coupled with the growing demand due to an increase in aging population, chronically ill patients and life expectancy, causing a rise in costs (Spatharou *et al*., 2020). It’s believed that the adoption of artificial intelligence (AI) can solve these problems and lead to a reduction in HC professionals’ burnout, timely detection of at-risk patients and save lives (Jiang *et al*., 2017). It can support the analysis of data, diagnoses and treatments and lead to a more preventive and precision medicine (Kaplan and Haenlein, 2019; Kumar *et al*., 2021; Liu *et al*., 2020; Paschen *et al*., 2020a, b), contributing to organizational flexibility and capabilities for the design and customization of customers’ needs (Kumar *et al*., 2023a, b).

Previous studies have investigated the impacts of digital technologies on BMs in marketing, media and automotive industries (Kumar *et al*., 2021; Reinartz *et al*., 2019; Verhoef and Bijmolt, 2019) and why they are implemented in the HC industry (Singh *et al*., 2024), but few investigated how the interaction between these technologies, in particular, AI-based, is changing the HC BMs and outcomes (Kraus *et al*., 2021; Laurenza *et al*., 2018). There is also a lack of consensus on the influence of AI in HC performance and the skepticism of HC professionals to adopt it (Raimo *et al*., 2022). It’s therefore relevant to conduct a thorough investigation into the influence of AI on HC BM. Therefore, this study aims to fill this gap by exploring how the adoption of AI can impact HC BMs and outcomes, as well as the ethical considerations involved in its implementation. In a nutshell, we sought to answer the following questions:

How is AI transforming the HC BMs and outcomes?

What are the ethical considerations for AI adoption in HC?

To answer these questions, we employed an empirically exploratory research methodology based on open-ended essay questions designed on prolific.co and collected data from 29 HC professionals with experience in AI, using purposive and snowball sampling techniques, from Europe and America in May 2024. We analyzed these data thematically using content analysis, and the findings revealed six effects of AI on HC BMs and outcomes, which include better patient outcomes and experience, standardization, accessibility, cost-savings, automation and efficiency, and innovation and discoveries. The study has also identified three ethical considerations for AI adoption in HC, and these include data issues, bias issues and liability issues. Lastly, from the recommendations given by our participants, the study has offered three AI implementation strategies, which include continuous assessment and compliance with regulations, continuous education and training, and human expertise integration. With these results, this study has made significant theoretical and practical contributions to the literature by advancing the study of AI and BMs in HC, incorporating different and new perspectives across boundaries, expatiating on how AI can help HC to improve its competitive positioning through the transformation of its value creation, value delivery and value capture architecture. The study has also discussed the ethical considerations impeding the adoption of AI in HC and how they can be addressed to successfully and ethically implement AI in this industry.

The subsequent sections of this study are organized as follows: section 2 reports the brief literature review on the study of AI and BMs in HC, and section 3 contains the research methodology covering the design, data collection and analysis. Section 4 reports the findings of the study, while section 5 contains the discussion and conclusion. Lastly, section 6 reports the theoretical and practical implications, as well as the future research directions.

## Literature review

### Artificial intelligence and its applications in healthcare

AI has been defined differently by different scholars; however, its concept was first introduced in 1956 (McCarthy *et al*., 2006), representing a technology that imitates human behavior. It has also been defined as an agent or a system (Poole *et al*., 1998). That deals with the use of computers to mimic human cognitive abilities and perform tasks intelligently using algorithms. It learns from experience, performs human-like jobs and adjusts to new inputs (Venkatesan, 2018). It involves the transformation of services or processes into automation that depends on intelligent computer systems or robots managed by computers that function independently without human control (Copeland *et al*., 2019). AI uses machine learning (ML) and deep learning (DL) to execute tasks and facilitate operations. ML is the ability of a system to continuously improve its capabilities and perform tasks by continuously acquiring and analyzing data and information automatically to improve its performance (Murphy, 2012). ML uses other types of AI, such as natural language processing (NLP), voice technology and robotics (Chen and Decary, 2020). NLP is the ability to analyze human languages and allows the computer to recognize, interpret and manipulate human languages (Paschen *et al*., 2020a, b). While DL uses neural networks similar to human brain to discover complex structures in data by applying a backpropagation algorithm operating on multiple layers of abstraction (Esteva *et al*., 2019; LeCun *et al*., 2015). DL is usually applied in speech and image recognition (Schmidhuber, 2015). The HC sector applies ML and DL to predict, diagnose, interpret, learn and offer treatment recommendations for various types of diseases.

The use of AI in HC is eminent due to increased demand for tracking, tracing and identification of disease as well as for various other medical uses (Hee and Yoon, 2021). Studies have revealed that its applications in HC can process, analyze and interpret data and make decisions independently to realize organizational objectives (Kaplan and Haenlein, 2019; Liu *et al*., 2020; Paschen *et al*., 2020a, b). AI has been used in radiology for X-ray scans, lung nodule detection, brain scans, lesion detection and breast mass identification (Arbabshirani *et al*., 2018; Ginneken *et al*., 2015; Liew, 2018; Samala *et al*., 2016; Topol, 2019). Dermatologists use AI to classify cancer, detect mutations of genome drivers, detect metastatic breast cancer and classify brain tumors (Capper *et al*., 2018; Coudray *et al*., 2018; Hsieh, 2017; Yang *et al*., 2018; Yu *et al*., 2016). Ophthalmologists also use AI to grade and monitor diabetic retinopathy, glaucoma and age-related macular degeneration and analyze congenital cataracts (Abramoff *et al*., 2018; Kumar *et al*., 2023a, b; Long *et al*., 2017). It was applied to diagnose heart attacks, cardiovascular risk predictions, classify hypertrophic cardiomyopathy and screen electro-cardiac activities (Nikus *et al*., 2011; Weng *et al*., 2017; Zhang *et al*., 2018). Convolutional neural networks were also used to diagnose and analyze various skin malignancies and achieved accuracy equivalent to that of dermatologists (Esteva *et al*., 2017). AI has facilitated the design of individual treatment plans, using genomes and factors such as age, gender and ethnicity (Nam *et al*., 2019). It can speed up the analyses of data and aid clinical decisions, as well as provide early warnings for morbidity and mortality, and assist in the identification of abnormalities in laboratory test results (Ellahham *et al*., 2020; Sutton *et al*., 2020). However, despite the progress brought by this technology, its applications and adoption have been witnessing various challenges and resistance, including privacy, data sharing, accountability, ethics and trust (Kakale, 2023; Lee and Yoon, 2021; Lekadir *et al*., 2022; Manne and Kantheti, 2021). Hence, medical practitioners are reluctant to adopt digital technologies in the current processes (Pan *et al*., 2019; Smith *et al*., 2021). It’s therefore relevant to investigate the ethical considerations for AI adoption in HC to understand and provide ways to address them for wider acceptance and adoption of AI in this sector.

## Artificial intelligence and healthcare business models

Companies in different industries are transforming their strategies, BMs and value-creation processes using AI capabilities (Kulkov *et al*., 2021; Teece, 2018b). A firm’s BM entails its value architecture, how it’s created, delivered and captured (Teece, 2018a). Value creation is related to the value proposition and refers to the offer made by a firm to its customers (Shane and Venkataraman, 2001; Täuscher and Laudien, 2018; Teece, 2010; Yunus *et al*., 2010). The availability of resources, business structure and organizational flexibility are vital for value creation (Amit and Zott, 2001; Wirtz *et al*., 2010). Value delivery, on the other hand, deals with how the value created reaches the intended customers (Amit and Zott, 2001; Wirtz *et al*., 2010). While the value capture entails how a company converts the value created and delivered to customers into revenues (Abdelkafi and Täuscher, 2016; Baden-Fuller and Mangematin, 2013; Casadesus-Masanell and Zhu, 2010; Gambardella and AM McGahan, 2010). BM has also been conceived as the management of innovation and technology (Cutroneo *et al*., 2014; Dmitriev *et al*., 2014; Zott *et al*., 2011). BM spelled out structures that mix technological features and potentials and translate them into economic input that is used to satisfy markets and customers’ needs at a profit (Chesbrough and Rosenbloom, 2002). Digital technologies have been providing increasing opportunities to organizations and at the same time forcing managers to adapt and/or completely change their BMs to embrace new ones (Wirtz *et al*., 2010).

The use of digital technologies in HC can facilitate the development of new BMs due to their effects on work processes, leading to the improvement of productivity, patient experience and cost reduction (Yang and Hsiao, 2009). AI can facilitate the transformation of how HC services are delivered to patients by improving the quality of HC and efficiency in the use of medical resources (Lee and Lee, 2020; Yoon and Lee, 2019). It can also support the tailoring of treatment based on individual problems and needs (Bohr and Memarzadeh, 2020). AI, therefore, plays an important role in HC processes and can facilitate the creation, delivery and capturing of value for different stakeholders (Dal Mas *et al*., 2019; Dal Mas *et al*., 2020). Its application can improve patient outcomes and HC efficiency (Lebcir *et al*., 2021) as well as reduce clinical risks, costs and improve HC services (Ellahham *et al*., 2020; Secundo *et al*., 2019). The use of AI in this sector can facilitate efficient patient flow, enhance disease detection, reduce misinterpretation and diagnostic errors, as well as improve the efficiency of medical supply chains and HC delivery systems (Damoah *et al*., 2021; Fan *et al*., 2020; Kraus *et al*., 2021; Leone *et al*., 2021; Medtronic, 2018; Topol, 2019), and consequently lead to the optimization of resources application, cost management and HC treatment efficiency (Leone *et al*., 2021; Yu *et al*., 2018). AI can change HC from a traditional to a smarter system, affecting its entire value architecture and improving patient care and experience. Despite the growing interest in AI in this sector, there is no consensus on whether it influences HC performance (Raimo *et al*., 2022). And empirical studies in this area are limited (Choudhury and Asan, 2020). Consequently, conducting an empirical study to explore the implications of AI on HC BMs and outcomes is of paramount importance. Table 1 summarizes the existing literature on the relationships between AI and HC BMs.

**Table 1:** Existing literature on the impact of AI on healthcare business models and outcomes

Authors and year	Titles	Coverage	Methodology	Key findings
Singh *et al*. (2024)	Technological paradoxes and artificial intelligence implementation in healthcare. An application of paradox theory	Global	Open-ended essay questions (exploratory)	This study found ease of use, cost-effectiveness, accurate diagnosis and effective automation as the drivers of AI adoption in HC. The barriers include privacy, security, unsuitability for some illnesses, lack of education and financial constraints
Laudien *et al*. (2024)	Digital advancement and its effect on business model design: Qualitative-empirical insights	Global	Case study and semi-structured interview	Digital advancement enables value customization, modular design of offerings and real-time value creation
Dicuonzo *et al*. (2023)	Healthcare system: Moving forward with artificial intelligence	Local	Case study and semi-structured interview	AI supports the digitization of information and communication, connecting people to people and systems to people, empowering patients and system automation
Kulkov (2023)	Next-generation business models for artificial intelligence start-ups in the healthcare industry	Global	Case study and semi-structured interview	AI is helping companies improve their value creation by facilitating access to HC and privacy design. It reduces costs, provides faster services and reduces workload
Akinwale (2020)	Technology innovation and healthcare performance among healthcare organizations in Saudi Arabia: A structural equation model analysis	Local	Quantitative (structural equation modeling)	There are positive relationships between innovation efforts, technology innovation and HC performance. However, no correlation is found between HC performance and the age of the HC organization
Kumar *et al*. (2023a, b)	Artificial intelligence (AI)-enabled CRM capability in healthcare: The impact on service innovation	Local	Mixed method	AI contributes to organizational flexibility and capabilities, helping to customize and meet customers' needs. It indicates an indirect correlation between AI capabilities and innovation-based activities in firms
Pham *et al*. (2024)	Determinants and performance outcomes of artificial intelligence adoption: Evidence from US hospitals	Local	Quantitative	A huge market share can lead to AI adoption, but no correlation exists between the length of hospital stay and AI adoption. AI can increase inpatient and outpatient revenues and productivity but will not increase return on assets
Kakale (2023)	Of digital transformation in the healthcare (systematic review of the current state of the literature)	Global	Systematic literature review	HC digitization can increase organizational and managerial efficiencies, empower patients and enable remote disease monitoring. Unfavorable policies, culture and lack of skills, reliability and resources constrained digitization in HC
Pundziene *et al*. (2023)	Indirect effect of open innovation on clinical and economic value creation in digital healthcare: A comparative study of European countries	Local	Survey	The flow of knowledge and cooperation on innovation projects among internal units significantly impacts HC outcomes in terms of patient and physician experience in Lithuanian HC, while impacting only physician experience in Spanish HC. It can also increase clinical and economic outcomes
Tortorella *et al*. (2021)	Impacts of Healthcare 4.0 digital technologies on the resilience of hospitals	Global	Survey	Digital technologies can increase HC resilience to monitor, anticipate, respond and learn
Kraus *et al*. (2021b)	Digital transformation in healthcare: Analyzing the current state-of-research	Global	Systematic literature review	Digitization can lead to the transformation of HC BM, internal business efficiency, patient-centeredness, empowerment and interconnectivity among stakeholders
Laurenza *et al*. (2018)	The effect of digital technologies adoption in healthcare industry: A case-based analysis and business	Local	Case study and interview	Digitization can improve and ensure efficient HC business processes, quality, cost-effectiveness, automation and reduced response time
Von Wedel *et al*. (2022)	Effects of hospital digitization on clinical outcomes and patient satisfaction: Nationwide multiple regression analysis across German hospitals	Local	Survey	Digitization alone will not translate to patient satisfaction, the outcome of emergency care is positively affected by the user-perceived value of hospital digitization and easy access to electronic health records can lead to better elective surgery and could reduce death
Rachinger *et al*. (2019)	Digitalization and its influence on business model innovation	Local	Case study and interview	Digitization has strong effects on media and automotive industries and value creation. It also has effects on value proposition and value capture, leading to increased revenues
Angeli and Jaiswal (2016)	Business model innovation for inclusive healthcare delivery at the bottom of the pyramid	Local	Case study and interview	Business model innovative strategies that ensure inclusive healthcare delivery are cocreation of patient needs, community engagement, strategic partnerships, continuous involvement of customers, innovative medical technologies and economies of scale
Verhoef and Bijmolt (2019)	Marketing perspectives on digital business models: A framework and overview of the special issue	Global	Conceptual	Digital business models affect markets in terms of competition, globalization and sales, as well as firm performance in terms of shareholders' value, sales, profitability, brand and value equities
Tortorella *et al*. (2022)	Contributions of Healthcare 4.0 digital applications to the resilience of healthcare organizations during the COVID-19 outbreak	Local	Case study and interview	HC 4.0 contributes to the resilience of hospitals in Brazil during COVID-19. They support their supply chain, diagnosis, treatment and follow-ups in terms of monitoring, anticipation, responding and learning

**Note(s):** CRM: Customer Relationship Management

**Source(s):** Authors’ own work

## Theoretical lens (resource-based view theory)

Resource-based view (RBV) theory posits that firms’ resources have a direct correlation with their performance (Barney, 1991; Chatterjee and Kulkarni, 2021). It states that organizational resources are any tangible or intangible assets owned by an organization (Mikalef and Gupta, 2021). These resources could include all forms of assets, processes, dynamic capabilities, information, attributes and knowledge used by an organization to develop and implement ideas and/or strategies designed to increase its efficiency and performance (Barney, 2001). The theory also stresses the importance of the rareness, inimitability and irreplaceability of such resources for creating superior value to improve the performance and competitive advantage of a firm (Barney, 2001; Ghasemaghaei, 2021). Studies found the existence of relationships between RBV, technological innovation and organizational performance (Akinwale, 2020; Dias *et al*., 2021; Fullerton and Wempe, 2009; Gupta and George, 2016). For example, AI has the potential to bring competitive advantages to businesses (Chaudhuri *et al*., 2021). Its capabilities are becoming increasingly important resources for business advancement and performance improvement (Belhadi *et al*., 2024; Lou and Wu, 2021; Mikalef and Gupta, 2021). Companies are implementing it to transform their BM and create value (Kulkov *et al*., 2021; Teece, 2018b).

This study, therefore, considers RBV theory important and relevant to understanding how HC BMs and outcomes can be transformed using AI technology. This theory was applied to investigate the relationship between technology innovation and HC performance in Saudi Arabia (Akinwale, 2020). Scholars have explored how the application of new technology, such as AI, can impact the BMs of companies (Kumar *et al*., 2021; Reinartz *et al*., 2019; Verhoef and Bijmolt, 2019). For example, Dicuonzo *et al*. (2023) studied how AI was applied at Humber River Hospital to effectively manage the digitization of information and communication, connecting people to people and system to people. Their findings revealed that the adoption of AI can empower patients, lead to system automation, increase bed capacity, reduce errors and save time. The study of MSD Italy, a subsidiary of US-based company Merck & Co., Inc., revealed that the adoption of digital technologies can improve HC business processes, efficiency and quality as well as lead to the automation of communication, monitoring, diagnosis and treatment (Laurenza *et al*., 2018). Application of AI in HC start-ups can assist companies to improve their value creation, accessibility and privacy design; it can also improve diagnosis, decision support and drug discovery while enabling faster HC services, reducing costs and easing workload (Kulkov, 2023). A study of the technological paradoxes and AI implementation in HC revealed ease of use, cost-effectiveness, accurate diagnosis and effectiveness of automation as the drivers of AI adoption in HC. Findings from this study also revealed privacy, security unsuitability for certain clinical illnesses, lack of skills, education and resistance as the barriers preventing the implementation of AI in HC (Singh *et al*., 2024).

A study of the relationship between technology innovation and HC performance was carried out in Saudi Arabia (Akinwale, 2020). The results showed that innovation efforts can increase technological innovation and HC performance. Another study of the HC professionals’ perceptions on the indirect effect of open innovation on clinical and economic value creation was carried out in Spain and Lithuania. The results revealed that open innovation indirectly affects clinical and economic value creation through patient and physician experience; these results, however, showed that open innovation is context-dependent and differs between countries (Pundziene *et al*., 2023). Digital technologies can also increase the resiliency of hospitals to monitor, anticipate, respond and learn, including during COVID-19 (Tortorella *et al*., 2021, 2022). Another study of digitization and its effects on clinical outcomes and patient satisfaction was carried out in German hospitals; the results showed that the adoption of digital technologies alone will not lead to patient satisfaction but the value of that digitization would. The study stressed the importance of the user-perceived value of the digitization and ease in accessing electronic health records in facilitating better elective surgery and emergency care (Von Wedel *et al*., 2022). Meanwhile, Pham *et al*. (2024) explore the determinants and outcomes of AI adoption in the US hospitals. Their findings revealed that huge capital outlay can lead to the adoption of AI, but the average length of stay does not correlate with AI adoption. This study also showed that AI adoption can increase the total inpatient and outpatient revenues. While many studies have examined the application of digital technologies in HC, businesses and their effects on BMs in marketing, media and automotive industries (Kumar *et al*., 2021; Reinartz *et al*., 2019; Verhoef and Bijmolt, 2019), few have explored their implications on HC BMs using empirical methods (Kulkov, 2023; Laurenza *et al*., 2018; Singh *et al*., 2024), in particular, AI-based technologies, coupled with a lack of consensus on whether AI influences HC performance (Raimo *et al*., 2022) and the reluctance of HC professionals to adopt it (Pan *et al*., 2019; Smith *et al*., 2021). Our study aims to fill these gaps by exploring how the adoption of AI can impact HC BMs and outcomes, using RBV and the ethical considerations involved in its implementation. We believe that studying the correlation between AI and HC BMs through the lens of RBV will discover new perspectives and contribute to the literature.

## Methodology

To understand how AI is impacting HC BMs and outcomes, this study employs an exploratory research methodology (Swedberg, 2020). The objective of using this method is to solicit enough data and information from HC professionals to understand their views concerning the possible changes and progress that the adoption of AI in HC could have on processes, operations, services, products and outcomes. The exploratory research method is beneficial for collecting initial data, making clear patterns and themes. This approach is also useful in a novel and underinvestigated research context (Busetto *et al*., 2020). Qualitative research helps and enables a researcher to discover and understand processes that are hidden within an organization (Bluhm *et al*., 2011). This methodology is relevant to our research as it helps us explore literature extensively to answer our research questions. In this context, our study employs open-ended essay questions to collect data (Singh *et al*., 2024). Using open-ended questions can facilitate the collection of qualitative data or views of respondents and is popular in management research (Dhir *et al*., 2017). This approach supports our data collection from different respondents with diverse experience and perspectives (Chaudhary *et al*., 2022). Meanwhile, to facilitate the appropriate application of this method, and collect enough relevant data, we targeted HC systems in Europe and America because of the growing HC challenges, aging population and chronic diseases (European Commission, 2020). Lack of HC personnel (Michel and Ecarnot, 2020), inefficiency (OECD, 2017) and inequalities (Hamed *et al*., 2020) as well as users’ mistrust to adopting AI-based technologies in HC (Li *et al*., 2021). The use of this approach in this study is therefore relevant.

## Data collection approach

Our targets were HC professionals and experts who have experience in using AI from different and diverse HC sectors and departments. A purposive sampling technique was adopted to recruit a sample of over 70 HC professionals and experts, such as doctors, nurses, pharmacists, HC consultants, psychologists, business analysts, medical engineers and other relevant experts. These professionals were invited to participate in this study by filling in open-ended essay questions designed on prolific.co in May 2024. The aim is to solicit the opinions of these experts about AI adoption in HC. The criteria for inclusion were designed to include only participants who are aware of AI technology and have been using it in their respective jobs at least twice per week. This is to allow the inclusion of only participants with a great deal of knowledge about the application of AI in HC and ready to voice their experience and opinions. Similarly, a snowball sampling technique, where participants referred and invited other experts in the field, was applied (Christofi *et al*., 2023). In total, 29 participants matched our inclusion criteria, and they were selected and included in this study (Malodia *et al*., 2023). The data collection processes were valid and rigorous, considering our respondents’ background, departments, locations, sectors, and diverse knowledge and experience (Christofi *et al*., 2023; Malodia *et al*., 2023). Meanwhile, the data were collected using open-ended essay questions (Chaudhary *et al*., 2022).

The structure of participants who responded to our requests was 58.6% females and 41.4% males, out of which 27.59% were from the United States, 41.38% were from the United Kingdom, 13.79% were from Italy, 6.89% were from Canada, 6.89% were from Sweden and 3.45% were from Belgium. They were all from diverse HC organizations, medical domains, sectors and departments, ranging from emergency departments, pharmacy, radiology, mental health, medicine, engineering and consulting to researchers and many other areas of medical profession, with diverse ages and work experience. Table 2 summarizes the demographic information of these participants. The composition of these participants allowed us to gather different perspectives and understandings of how the integration of AI across the healthcare sectors and departments of HC is transforming the creation, delivery and capture of value. This has also improved our understanding of the benefits, limitations and challenges of AI adoption in HC and enhanced the relevance, quality, trustworthiness and generalizability of this study.

**Table 2:** Demographic details of the respondents

Respondent details	Frequency	Percentage (%)
*Gender*		
Male	12	41.4
Female	17	58.6
*Age*		
Less than 25 years	5	17.24
26–35 years	10	34.48
Over 35 years	14	48.28
*Occupation*		
Emergency medical services	3	10.34
Nurse	6	20.69
Pharmacist	3	10.34
Psychologist	1	3.45
Paramedic	1	3.45
Doctor	3	10.34
PhD	1	3.45
Healthcare director	1	3.45
Medical engineer	2	6.89
Business analyst	1	3.45
Healthcare consultant	2	6.89
Others	5	17.24
*Experience (* *y* *ears)*		
3–5	8	27.59
5–10	12	41.38
10–20	5	17.24
More than 20	4	13.79

**Source(s):** Authors’ own work

The open-ended essay questions were developed after an extensive literature review (Dicuonzo *et al*., 2023; Singh *et al*., 2024) and our experience in the field. These questions were designed to explore how AI is impacting HC BM and outcomes, the ethical considerations involved in its implementation and how it can successfully be implemented in HC. Respondents were asked to give their general understanding of AI in HC, the motivation behind its adoption, how it changes tasks and operations, how it affects products and services offered to patients, how it changes the way these products and services are delivered to patients, how it is impacting outcomes and performance and what its ethical considerations are and finally to give their opinions on its implementation strategies in HC. To improve the quality of this study and ensure that we are asking the right questions, four HC professionals and experts in digital transformations were consulted and asked to verify and give their opinions on the proposed questions. We compared their observations and implemented necessary changes, elimination, and adaptations; eight questions were finalized and used in this study. In addition, we conducted pilot testing on prolific.co using five essays to ensure the smoothness, validity and reliability of the data collection processes. Having encountered no challenges, we proceeded with the distribution of additional 24 essays, bringing the total to 29 essays. All the participants received a link leading to a form containing questions asking their opinions on AI in HC; they filled in those forms and then returned them.

## Data analysis

A content analysis approach was used for data analysis (Weber, 1990), using recommendations (Gioia *et al.*, 2013). The completed forms were collected, organized, categorized and thematically arranged for a broader understanding of the data. This procedure helped us develop a data structure that outlined the first-order, second-order and aggregate dimensions of the study. The analysis processes involved using informants’ terms, excerpts and statements to develop codes and categories, which were then distilled into abstract themes and aggregate dimensions. To ensure the reliability and validity of the analysis, these processes were carried out by two independent researchers; their work was compared for further assessments of possible misinterpretation and reliability issues (Miles and Huberman, 1994). Consequently, no conflict or issues were found; hence, we can attest to the validity, transparency and reliability of the analysis procedure.

As we intend to explore how AI is affecting HC BMs and outcomes, we found Gioia *et al.*’s (2013) recommendations for analyzing qualitative data, suitable for our current problems. Following those guidelines, we developed 100 zero-order codes by reading and analyzing respondents’ scripts line by line and then relating them to a specific research question (e.g. the question about how AI is changing the way tasks/operations are conducted in HC: “it will make tasks quicker and easier to do”, streamlined administrative processes). These codes were then synthesized and converted into 55 first-order codes, considering their dissimilarities and interconnections (e.g. “it fosters a more patient-centric HC system”, “enabling patient recovery rates and overall HC management”). First-order codes were then further distilled into 30 second-order themes by clustering and categorizing them into more meaningful themes (e.g. “money and time savings”, “it could reduce staffing”). The second-order themes were further analyzed and distilled into 12 aggregate dimensions, in which each dimension served as the key finding of a particular theme (e.g. cost-savings, data issues), representing the final findings of the study and the completion of the analysis procedure.

## Findings

The underpinning theory of this study is the RBV, which asserts the importance of organizational resources in helping it achieve high performance (Barney, 1991; Chatterjee and Kulkarni, 2021); these resources could be any tangible or intangible assets (Mikalef and Gupta, 2021). We adopted this theory as it helped us understand, through different views and perspectives of HC professionals, how AI is reshaping HC BMs and impacting outcomes, as well as the ethical considerations involved in its implementation. Table 3 presents the data structure of the analysis. This study has identified six effects of AI on HC BMs and outcomes that include better patient outcomes and experience (Kulkov, 2023; Lebcir *et al*., 2021), standardization (Kulkov, 2023; Laurenza *et al*., 2018), accessibility (Kakale, 2023), cost savings (Laï *et al*., 2020; Pham *et al*., 2024), automation and efficiency (Kraus *et al*., 2021; Reddy, Allan and Coghlan, 2020) and innovation and discoveries (Akinwale, 2020; Kulkov, 2023). Moreover, three ethical considerations for AI adoption in HC include data issues (Singh *et al*., 2024), bias issues (Mahadevaiah *et al*., 2019; Stone, 2018) and liability issues (Lee and Lee, 2020). Lastly, the study has identified three AI implementation strategies based on recommendations given by our respondents, which include continuous assessment and compliance with regulations (Mahadevaiah *et al*., 2019; Seddon and Currie, 2013), continuous education and training (Lee and Lim, 2021) and human expertise integration (Angeli and Jaiswal, 2016).

**Table 3:** Data structure

First order concepts	Second-order themes	Aggregate dimensions
-Better patient outcomes and improved healthcare performance -Reduced anxiety for patient -It furthers patient care and regrettably increases profit -Enabling patient recovery rates and overall healthcare management -Patient support is better -It fosters a more patient-centric healthcare system	Enhance diagnostics Patient-centric healthcare system Patient support is better Further patient care	Better patient outcomes and experience
-It will provide much-needed standardization -Consistency in information provided to patients -Postoperative care will be standardized based on the latest research and guidance -Unbiased support and consistency in outcomes	Consistency in information Standardization Use of latest research Accuracy and less subjectivity	Standardization
-It could also be accessible, having something online/on the phone, such as e-consent -Facilitate remote monitoring and telemedicine -It is less personal and interactive; however, it's efficient	Remote monitoring and telemedicine Accessibility	Accessibility
-It could reduce staffing, decrease wait time and lower costs -AI provided to offset the shortfall of nurses and doctors -A way to get around poor recruitment and retention -Money-saving and time-saving	Money and time savings It could reduce staffing A way to get around poor recruitment and retention	Cost-savings
-It can streamline patient information care -AI optimizes workflow while keeping fair and balanced -Optimize healthcare operations -It improves efficiency, accuracy and accessibility -It delivers them faster and possibly with some accuracy/less subjectivity -Waiting lists have come down in mental health due to AI completing referrals/screening checks -Streamlined administrative processes -It will make tasks quicker and easier to do -AI is revolutionizing healthcare operations by automating tasks, enhancing diagnostics, enabling remote care, optimizing resource allocation and accelerating drug discovery -Delivering focused on objective processes -It's addressing operational challenges	Optimizes workflow Streamlined administrative processes Make tasks quicker and easier to do	Automation and efficiency
-Improved diagnostic tools -Treatments and antibiotic choices will be based on the latest research -Products may be cheaper -It's driving medical innovation -It can support clinical as well as research initiatives	Improved diagnostic tools Driving medical innovation Accelerating drug discovery Supporting clinical and research initiatives	Innovation and discoveries
-Information consent is absolutely necessary -Mishandling of personal information (PI) is a big problem and concern right now -Data breaches -Data protection and privacy -It's at the risk of being accessed by other people -I feel privacy is a big ethical consideration as confidentiality of patient data should be ensured -Safety -Security	Data breaches	Data issues
-Mitigating biases in algorithms	Biases in algorithms	Bias issues
-Navigating liability issues is critical -I don't think they will change much -I think it has the possibility to increase efficiency, but it is not having huge outcomes on performance as of right now	Liability issues	Liability issues
-It would need to have a failsafe built in to ensure that it works 100% -Addressing biases ensures fair access to healthcare for all -AI should still comply with laws and regulations -Transparency and accountability build trust in AI -AI needs to become more sophisticated if the public is to trust the information provided by it	Need to have a failsafe built in Address biases Comply with regulations	Continuous assessment, modification and compliance with regulations
-Ongoing education and training of healthcare professionals are vital in the adoption of AI in healthcare -Education on ethical considerations	Ongoing education and training	Continuous education and training
-Doctors should always be the ones making the final decisions -Colleagues have less autonomy since machines are telling us what to do and when to do it -In the future, people will be in the hands of automated services; however, this could result in misdiagnoses and inaccurate assessment	Final decisions should be made by the doctors	Human expertise integration

**Source(s):** Authors’ own work

## Effects of AI on healthcare business models and outcomes

### Better patient outcomes and experience

**Figure 1:**
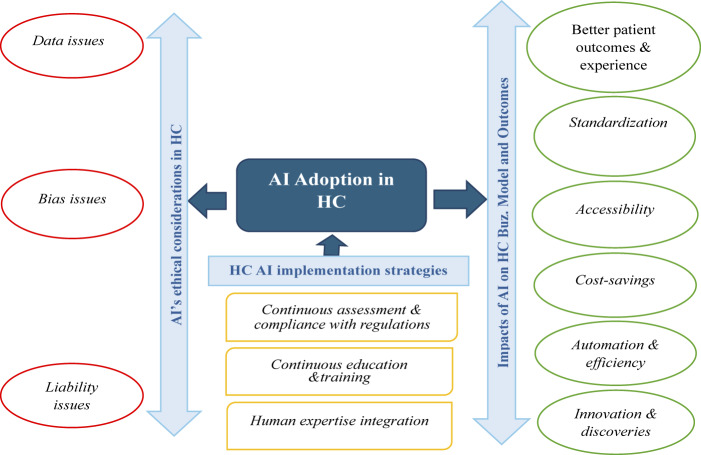
Summary of the findings. **Source(s)**: Authors’ own work

The adoption of AI in HC is improving patients’ outcomes and experience by facilitating early disease detection, enhancing diagnoses, treatment, faster recovery, decision supports and engagement. AI can support disease forecasting, diagnosis and treatments that are useful for both patients and physicians (Reddy, 2018). Many scholars have stated the relevance of AI in furthering patient care. For example, the integration of AI in clinical decision support has facilitated different diagnoses, discovered early signs of morbidity and mortality, as well as identified abnormalities in laboratory tests and radiological images (Sutton *et al*., 2020). Our findings revealed that AI adoption can personalize, improve and better patient care and support, improving their outcomes and experience (Kumar *et al*., 2023a, b; Pundziene *et al*., 2022). Figure 1 contains the summaries of these findings. Our respondents have confirmed that AI is revolutionizing patient care through advanced diagnostic tools, such as International Business Machine (IBM) Watson for Oncology, da Vinci for minimally invasive surgeries, resulting in faster recovery, use of personalized medicine to tailor treatments based on individual needs, and application of predictive models and algorithms for proactive and preventive medicine, all designed to further patient care and experience. This technology will continue to improve patients’ care as their demands and needs grow. Consider the following passages from our participants:

The integration of AI in healthcare is revolutionizing patient care by offering advanced diagnostic tools like IBM Watson for Oncology and minimally invasive treatments through robotic surgery systems such as the da Vinci surgical system, leading to faster recoveries and reduced risks. AI-powered remote monitoring devices and telemedicine platforms like Teladoc Health improve convenience and accessibility, especially for underserved areas. Personalized medicine is achievable as AI tailors treatment plans based on genetic profiles and treatment responses, while predictive analytics models enable proactive preventive care by identifying high-risk patients. Overall, AI adoption in healthcare enhances patient care experiences and fosters a more patient-centric healthcare system.

(P18, Female, 34, Italy, Doctor, Healthcare and Social Assistance)

AI in healthcare enhances diagnostics and treatment precision. For example, AI-driven imaging can identify tumors earlier, and personalized medicine algorithms optimize drug dosages for individual patients, improving outcomes.

(P8, Female, 42, Belgium, Pharmacist, Healthcare and Social Assistance)

## Standardization

Standardization in this context entails the degree of quality services, information, products and care delivered to patients because of AI adoption. The capacity to process information not only furthers the quality of patient care but also optimizes the management and operations of hospitals through the application of advanced knowledge and relevant technology (Srivastava and Singh, 2021). Therefore, AI adoption can enormously support an organization’s information-processing capability (Kaplan and Haenlein, 2019). Hospitals that adopt processing-actuation technologies like AI showed better operational performance (Tortorella *et al*., 2022) and improved and high-quality medical images (Pesapane *et al*., 2018). AI-based technologies facilitate the reduction of errors made by human judgment and ensure the administration of less subjective care (Uzialko, 2020). AI can identify and eliminate prescription errors or adverse events, thereby ensuring consistency and efficiency of medical care (Saddler *et al*., 2020). Our findings indicate that adoption of AI in HC can provide best practices and assurance that the services and information offered to patients are based on latest research and guidelines, which will lead to the provision of unbiased, standardized and consistent information and services. Take a look at the following excerpts from our participants:

It provides much-needed standardization and clear assurance that any services offered are current and factually based.

(P26, Male, 37, the United States, Healthcare Consultant, Medical/Healthcare)

Consistency in the information provided to patients, for example, post-operative care will be standardized and based on the latest research and guidance, such as NICE.

(P2, Female, 61, United Kingdom, Nurse, Healthcare and Social Assistance)

Provision of unbiased support and consistency in outcomes and experience.

(P21, Male, 30, United Kingdom, Emergency Medical Services, Healthcare and Social Assistance)

## Accessibility

Accessibility refers to the process whereby patients can obtain medical care and HC services anywhere they are and at any time. This has been achievable using AI-based monitoring devices to monitor and seek medical assistance. Telemedicine can allow physicians to evaluate patients’ medical conditions and administer treatments (Hu, 2003; Lin, 2012; Whitten, 2006). Our research discovered that the adoption of AI could increase accessibility to the HC through the digitization of communication and information, connecting people with people and systems with people, empowering patients and ensuring automation (Dicuonzo *et al*., 2023), hereby allowing people with rare diseases and underserved areas to access and obtain medical care and assistance. AI also facilitates the creation of value by companies through improved accessibility to HC (Kulkov *et al*., 2021), enabling and facilitating remote disease monitoring, management and accessibility to quality care (Kakale, 2023). The use of AI-based telemedicine also facilitates postoperative care through video conferencing, reducing pressure on both patients and doctors (Asiri *et al*., 2018; Parajuli and Doneys, 2017; Rao and Lombardi, 2009). These have been confirmed by our participants, who asserted that AI has significantly transformed HC, allowing the use of telemedicine and advanced medical imaging tools, such as X-rays, MRIs, CT scans and other monitoring devices to facilitate accessibility to care and improve patient outcomes. Consider the following passages:

AI adoption in healthcare significantly changes the services and products offered to patients in several ways: (1) improved diagnostic tools (can detect anomalies in medical images like X-rays, MRIs and CT scans with high accuracy); (2) enhanced remote monitoring (smartwatch and so on) and (3) optimized healthcare operations (such as appointment scheduling).

(P17, Female, 28, Canada, Nurse, Medical /Healthcare/Healthcare)

AI profoundly impacts healthcare outcomes by enhancing diagnostic accuracy, enabling personalized treatment plans, improving efficiency through automation, facilitating remote monitoring and telemedicine, enabling proactive preventive care and accelerating drug discovery. These advancements ultimately lead to better patient outcomes and improved healthcare performance.

(P18, Female, 34, Italy, Doctor, Healthcare and Social Assistance)

It could be easier to explain/show patients the results or outcomes, for example, if wanting to show how an operation works for this specific patient with their background that could be incorporated into the AI model, and the patient can see their risks/outcomes. It can also be accessible; having something online/on the phone, such as e-consent, is easier to access for most people.

(P16, Female, 29, United Kingdom, Doctor, Medical/Healthcare)

## Cost-savings

Adoption of AI in HC brings about cost-savings in terms of staff reduction, using predictive models to identify and track at-risk patients, well-tested tools for research and drug discovery, remote monitoring, and reduction in the readmission rates and reduction in the need to travel for medical care. AI-supported smart robots can perform operations and facilitate physicians’ work by diagnosing and recommending treatment plans, ensuring cost and time reduction (Lee, 2018). For example, the use of AI tools to identify at-risk patients in public hospital in the USA resulted in a readmission rate reduction and $4 million in savings in two years (Miyashita and Brandy, 2019). The findings of this study showed that the adoption of AI-based telemedicine can reduce the need to travel for medical intervention, leading to time and cost savings (Gunter *et al*., 2016). It can also lead to increase in inpatient and outpatient revenues and productivity (Pham *et al*., 2024) and cost-effectiveness (Laurenza *et al*., 2018; Pak *et al*., 2009). Many of our participants have confirmed the effectiveness of AI and its relevance for cost and time reduction in HC. Particularly, they stressed its importance in reducing the number of staff and the need for patients to enter HC facilities for medical intervention. It can help HC organizations solve problems associated with poor recruitment and retention and address issues related to the shortage of nurses and doctors. Look at the following statements:

It could reduce staffing and unnecessary travel, decrease waits and lower costs.

(P22, Male, 32, United Kingdom, PhD, Healthcare Researcher)

I think it would reduce the number of physical staff that need to be present and possibly reduce the need for patients to enter a healthcare facility as statistics could be monitored remotely which may reduce anxiety for patients.

(P3, Female, 36, the United States, Pharmacist, Healthcare and Social Assistance)

Be more effective and reduce the cost of healthcare.

(P8, Female, 42, Belgium, Pharmacist, Healthcare and Social Assistance)

## Automation and efficiency

Automation refers to the process whereby machines are used to perform tasks, functions and/or processes without human intervention; efficiency, on the other hand, represents the extent to which how well resources, time and money are used while performing tasks. Kraus *et al*. (2020) found that HC digitization can automate tasks and processes, resulting in managerial and operational efficiencies. The automation of HC activities could streamline processes, leading to time and energy savings (Laï *et al*., 2020). It could involve the application of chatbots and/or nursing robots to improve efficiency (Lee and Yoon, 2021). AI can ensure the effectiveness of automation (Singh *et al*., 2024), contributing to organizational flexibility and capability to meet customer needs (Kumar *et al*., 2023a, b). Our participants indicated that AI could analyze large datasets faster and more accurately to predict risks and outcomes, facilitating personalized care. AI could ensure the automation of med dispensing using robots, creating more time for HC professionals to cater to other patients’ needs. They also confirmed that AI could streamline HC processes, leading to the optimization of HC activities and operations. Consider the following excerpts:

AI transforms healthcare by improving accuracy and efficiency. For instance, AI algorithms analyze vast data sets to predict patient risks and outcomes, enabling proactive and personalized treatment plans.

(P18, Female, 34, Italy, Doctor, Healthcare and Social Assistance)

There could be automated med dispensing robots to each patient room in a hospital setting, getting rid of the rounding nurses’ need to chart and dispense medicine.

(P16, Female, 47, the United States, Other, Healthcare/Medical)

AI can be a revolution and a significant aid for healthcare workers. It could be very useful in streamlining the work considerably.

(P17, Female, 28, Canada, Nurse, Medical/Healthcare)

## Innovation and discoveries

The adoption of AI in HC has facilitated innovation and discovery processes through new and improved diagnostic tools, new software, applications, platforms for HC services, new and enhanced clinical research facilities and drug development. Our study has revealed that AI adoption can improve the innovation capabilities of firms (Kumar *et al*., 2023a, b). It can also support companies by improving their value creation (Kulkov *et al*., 2021). Digitization can strongly affect value creation, value proposition and value capture, resulting in increased revenue (Rachinger *et al*., 2019). Our respondents have stated that AI can change the HC landscape for the better, by improving outcomes, addressing operational challenges, driving medical innovations and completely transforming how HC is delivered and experienced, leading to a more patient-centric approach. They also confirmed its importance in facilitating personalized care using predictive analytics platforms such as ClosedLoop.ai, diagnostic tools like IBM Watson, chatbots, robotics for minimally invasive surgeries, such as da Vinci, and its relevance for clinical and research activities. Take a look at the following passages from our respondents:

AI in healthcare is indeed a transformative force, changing how value is created and delivered to patients. It’s improving patient outcomes, addressing operational challenges and driving medical innovation, drug discoveries and development, ultimately leading to a more efficient, accessible and patient-centric healthcare system.

(P22, Male, 45, Germany, Business Analyst, Digital Transformation)

AI transforms healthcare by offering personalized treatment plans, remote monitoring, predictive analytics for preventive care, enhanced diagnostics and streamlined administrative processes. Examples include Tempus for personalized therapies, wearable devices like the Apple Watch, predictive analytics platforms like ClosedLoop.ai, AI diagnostic tools like IBM Watson and AI chatbots like Babylon Health. Overall, AI revolutionizes healthcare, delivering more efficient and patient-centric services and products for better outcomes.

(P18, Female, 34, Italy, Doctor, Healthcare and Social Assistance)

It can be a valuable aid in diagnosis and can support clinical as well as research activities.

(P17, Female, 28, Canada, Nurse, Medical/Healthcare)

## Ethical considerations

### Data issues

Data issues are ethical considerations encountered while accessing, processing and using patients' data. These issues deal with the privacy and security of patients’ data. Our findings showed that AI uses a lot of data, which raises privacy concerns about how these data could be collected and shared (Fernández-Cardeñosa *et al*., 2012; Rolim *et al*., 2010); patients fear losing control over their data (Ermakova *et al*., 2013). Privacy issues have exacerbated this fear, leading to mistrust and unwillingness to adopt AI-based technologies (Hee and Yoon, 2021; Vaio *et al*., 2020). Our participants stated the importance of ensuring the privacy and security of patients’ data and the relevance of obtaining informed consent before obtaining and using these data. They confirmed that data breaches and mishandling of personal information are big problems in HC; they called for a concerted effort to protect and safeguard patients’ data and interests, reaffirming that this will eradicate the patients’ fears and apprehensions and boost their confidence in accepting and adopting AI. Consider the following excerpts from our respondents:

Ethical considerations in AI healthcare include ensuring data privacy, mitigating bias in algorithms, maintaining human oversight, securing informed consent and prioritizing patient welfare to prevent disparities in care quality and accessibility.

(P8, Female, 42, Belgium, Pharmacist, Healthcare and Social Assistance)

Data breaches and mishandling of PI is a big problem and concern right now.

(P10, Male, 32, the United States, Pharmacist, Healthcare and Social Assistance)

I feel privacy is a big ethical consideration as confidentiality of patient data should be ensured.

(P12, Female, 22, United Kingdom, Psychologist, Healthcare and Social Assistance)

## Bias issues

Bias issues arise when AI discriminates and/or displays false results against certain individuals, groups or subgroups due to their nationality, skin color, gender, ethnicity and/or geographical location. These biases could be human-induced during the development processes of such an application or algorithm (Howard and Borenstein, 2018; Monteith and Glenn, 2016) or data-induced which occurs during the training processes of an algorithm because of underrepresentation or insufficiency of the data (Mooney and Pejaver, 2018; Senders *et al*., 2018). Bias can lead to varying results, such as false positives or negatives, and cause unintended harm or discrimination against certain categories of people or groups (Cirillo *et al*., 2020). These constitute mistrust and serious challenges for AI adoption in HC. Our participants stated the importance of mitigating and eliminating these biases to ensure fair and equitable access to HC services for all. Consider the following passages from our participants:

In the integration of AI into healthcare, ethical considerations are vital. Protecting patient data privacy and obtaining explicit consent for AI use is crucial. Addressing biases ensures fair access to healthcare for all. Transparency and accountability build trust in AI. Patients should have informed consent and autonomy in AI-assisted treatments. Healthcare professionals must ensure ethical AI implementation and fair resource allocation. Continuous monitoring of AI societal impacts is necessary. Collaboration among stakeholders is key for ethical AI adoption in healthcare.

(P18, Female, 34, Italy, Doctor, Healthcare and Social Assistance)

Ethical considerations in AI healthcare include ensuring data privacy, mitigating bias in algorithms, maintaining human oversight, securing informed consent and prioritizing patient welfare to prevent disparities in care quality and accessibility.

(P8, Female, 42, Belgium, Pharmacist, Healthcare and Social Assistance)

## Liability issues

The adoption of AI in HC faces responsibility attribution problems, causing widespread debates and concerns. The question on everyone’s mind is: who should be held liable in cases where AI causes harm to patients? This has put forward a test to the current practices concerning safety assurance and clinical accountability (Habli *et al*., 2020). In a recommendation report to the EC in 2016 on Civil Law Rules on Robotics, intelligent devices were given personality (electronic persons), making them liable for the harm they cause (Pellegrino, 2004). However, it was argued that AI doesn’t possess free will; hence, it cannot be granted a personality status (Sparrow, 2007; Ting *et al*., 2017); therefore, it cannot be a responsible subject (Coeckelbergh, 2020). Others argue that perhaps in the future, if AI becomes more advanced and human serves just as supervisors, then AI can be considered a legal subject (Weaver, 2019). Our participants think that it’s critical and important to ensure compliance with regulations and navigate liability challenges. They confirmed that ensuring transparency and accountability can build trust for AI adoption in HC. Consider the following excerpts from our respondents:

AI adoption in healthcare faces integration challenges with existing systems, requiring substantial updates and training. Ensuring regulatory compliance and navigating liability issues are critical. Additionally, AI may disrupt job roles, necessitating new skills for healthcare professionals, and care must be taken to ensure equitable access to prevent exacerbating healthcare disparities.

(P8, Female, 42, Belgium, Pharmacist, Healthcare and Social Assistance)

Transparency and accountability build trust in AI.

(P18, Female, 34, Italy, Doctor, Healthcare and Social Assistance)

## Implementation strategies

### Continuous assessment, modification and compliance with regulations

To successfully implement AI in HC, periodic audit and risk assessments of AI tools were recommended (Kaminski and Malgieri, 2021; Reisman *et al*., 2018), to include pre-market evaluation and post-market follow-ups, according to the Federal and Drug Administration (FDA) (FDA, 2022), to ensure the development of ethical and trustworthy AI solutions in medicine and HC (Lekadir, 2022). It is, however, recommended that to ensure the protection of patients’ interests, regulations such as medical device, data privacy and liability laws must be implemented (French-Mowat and Burnett, 2012; Jussupow *et al*., 2021; Ma *et al*., 2023) and modify the current legislation (Shi *et al*., 2020). Our respondents reaffirmed the importance of monitoring and tracking AI performance in HC after deployment, to ascertain its potential and correct any problems (Lekadir, 2022) and ensure compliance with regulations, adequate awareness, transparency and accountability to build trust for its wider acceptance and adoption. Consider the following passages from our participants:

It would need to have a fail-safe built-in to ensure that it works 100 percent every time. The patient would need to be aware that they are interacting with AI and not be fooled into thinking they are a real person. Informed consent is absolutely necessary. It needs to be not purely a money-saving exercise and needs to actually be proven to improve patient outcome and experience.

(P3, Female, 36, the United States, Pharmacist, Healthcare and Social Assistance)

AI should still comply with laws and regulations, especially in a delicate area like Healthcare which deals with human lives.

(P10, Female, 23, the United States, Doctor, Healthcare and Social Assistance)

## Continuous education and training

To successfully implement AI in HC, there is a need to train and educate HC professionals and patients, to equip them with skills on how to operate, understand and interpret AI results, informing them of its benefits, risks and limitations (FDA, 2022). The education and training programs should include knowledge of AI structures, input data requirements and interpretation of its results (Shi *et al*., 2020). Technical skills of HC AI operators also need to be upgraded and updated for its smooth operations. There is also a need to increase the literacy level of HC AI users; this led to the call for an update of educational curricula and programs in medicine as well as to increase interdisciplinary orientation, including the organization of lectures, seminars and workshops, designed to impart all the necessary knowledge, skills and training to HC AI users (McCoy *et al*., 2020; Rampton *et al*., 2020). Our respondents confirmed that as AI evolves, there is a need for HC professionals to stay up to date on the latest AI development, applications and interpretation of its results to maintain best practices, facilitate its ethical application and maximize its benefits. Consider the following passages from our participants:

Ongoing education and training of healthcare professionals are vital in the adoption of AI in healthcare. As AI technologies evolve, healthcare providers must stay updated on the latest developments and best practices. This includes training on effectively utilizing AI tools, interpreting AI-generated insights and maintaining patient trust. Education on ethical considerations, data privacy and regulatory compliance is also crucial. By investing in education and training initiatives, healthcare organizations can maximize the benefits of AI adoption while ensuring responsible and ethical use.

(P18, Female, 34, Italy, Doctor, Healthcare and Social Assistance)

AI adoption in healthcare faces integration challenges with existing systems, requiring substantial updates and training. Ensuring regulatory compliance and navigating liability issues are critical. Additionally, AI may disrupt job roles, necessitating new skills for healthcare professionals, and care must be taken to ensure equitable access to prevent exacerbating healthcare disparities.

(P3, Female, 36, the United States, Pharmacist, Healthcare and Social Assistance)

## Human expertise integration

This study recommends that human expertise needs to be involved and integrated into AI to ensure the development of HC AI algorithms based on cocreation (Leone *et al*., 2021), maintaining strong collaboration at every stage of AI design, development, validation and deployment in the HC (Filice and Ratwani, 2020), to improve algorithms’ credibility and performance, generalizability and acceptability by experts (Luo *et al*., 2021; Sun *et al*., 2022). A strong engagement with various HC stakeholders will encourage future AI to consider maintaining a close interaction between end users and algorithms, otherwise called human–computer interaction (Xu, 2019). This will increase and optimize the performance, adoption, early detection and correction of any harmful features in the HC AI tools and system. Multi-stakeholder engagement in this area will ensure a balanced approach to incorporating safety, quality, equity, fairness, transparency, traceability, accountability and ethical features, needs and requirements in medical AI tools, while at the same time increasing the explainability and acceptability of these tools in HC. Our respondents warned against over-reliance on AI, stressing that it can exacerbate biases and misdiagnosis issues. They encouraged human involvement to oversee AI processes, for accurate diagnosis, interpretation and decision-making, to ensure its fair, ethical and transparent application in HC. Take a look at the following excerpts from our participants:

AI services are provided to offset the shortfall of nurses and doctors. In the future, people’s health will be in the hands of automated services. This could result in misdiagnoses and inaccurate assessments. For example, the robot may not understand a particular phrase or misinterpret what the patient is saying. Therefore, humans will still be required to step in when needed.

(P4, Female, 39, United Kingdom, Nurse, Healthcare and Social Assistance)

What is and what is not an AI application’s job to do? Where and who decides the limit?

(P2, Female, 61, United Kingdom, Nurse, Healthcare and Social Assistance)

## Discussion and conclusion

This study explores the impacts of AI on HC BMs and outcomes using a RBV theory (Barney, 1991; Chatterjee and Kulkarni, 2021). This theory emphasizes the importance of both tangible and intangible organizational resources in enabling the creation of high-value products and services, thereby enhancing performance and achieving competitive advantages. Using RBV in this research is relevant as it helps us explore and understand the implications of AI adoption on HC BMs and outcomes, its ethical considerations and implementation strategies. Using open-ended qualitative essay questions, this study inductively collected and analyzed the views of HC professionals and identified six effects of AI on HC BMs and outcomes, three ethical considerations and three implementation strategies. As regards the implications of AI adoption on HC BMs and outcomes, this study revealed that using AI in HC can lead to better patient outcomes and experience, by ensuring faster and accurate disease detection, diagnosis and treatment. Enabling the implementation of advanced AI tools and equipment, such as IBM Watson, da Vinci surgical system, Teladoc., these results confirmed the findings (Reddy, 2018; Kumar *et al*., 2023a, b; Pundziene *et al*., 2022). The RBV has therefore extended and improved our understanding of how tangible and intangible AI resources can be used to create high-value HC products and services to improve patients’ experience and outcomes.

The adoption of AI can lead to the standardization of HC products, services, and information; consequently, improving the HC quality. Using AI in HC can ensure implementation of best practices, latest research capabilities, and guidelines, improving hospitals' operational and processing capacity, as well as facilitating the provision of consistent, standardized, unbiased, and quality HC to patients (Kaplan and Haenlein, 2019; Srivastava and Singh, 2021), reduce errors induced by human judgment (Uzialko, 2020), such as prescriptions (Saddler *et al*., 2020), thereby improving operational performance (Tortorella *et al*., 2022); contrary to the findings (Raimo *et al*., 2022) that asserted a lack of consensus on the influence of AI on HC performance. Our study also revealed the relevance of AI in this industry in increasing accessibility to quality care, particularly for underserved areas and people with rare diseases. The application of advanced monitoring devices, video conferences and imaging systems, such as X-rays, MRIs and CT scans, can facilitate remote diagnoses to accurately detect and treat malignancies, without the need for physician–patient contact, increasing access to HC and saving time and money (Kakale, 2023; Kulkov *et al*., 2021). AI capabilities, therefore, served as very important organizational assets in facilitating accessibility to high-quality care, impacting HC BMs and influencing performance.

High costs of obtaining medical care have been hindering the affordability and effectiveness of many HC organizations around the globe; our study revealed that implementation of advanced predictive models to identify at-risk patients, devices for remote monitoring, latest research tools for drug discovery and reduction of inpatients and the number of HC staff will reduce time and costs for both patients and HC organizations (Lee, 2018; Pham *et al*., 2024). These results were also confirmed by Gunter *et al*. (2016) and Miyashita and Brandy (2019). The adoption of these tools will ensure HC cost-effectiveness (Laurenza *et al*., 2018; Pak *et al*., 2009). AI can automate operations and bring about efficiency in the HC; our findings showed that AI can analyze large amounts of data, dispense medicine and perform operations, thereby automating and streamlining tasks and operations, improving efficiency, leading to increased operational capacity and organizational flexibility in the HC (Kumar *et al*., 2023a, b; Singh *et al*., 2024). AI implementation in HC can revolutionize and disrupt innovation and discoveries in this sector, leading to the creation of improved medical devices and tools, reconfiguring HC BMs and the value creation processes. This study has demonstrated how AI has facilitated the introduction and application of advanced machines and tools, such as IBM Watson, chatbots, monitoring, predictive analytics and data analytics in the HC, improving and leading to a more patient-centric approach, minimally invasive surgeries, quality diagnoses and treatments (Kumar *et al*., 2023a, b), revealing the relevance and importance of AI in transforming and reshaping innovation and creativity in the HC industry.

As regards the ethical considerations for AI adoption in HC, our study confirms the findings (Hee and Yoon, 2021; Vaio *et al*., 2020) related to data concerns as one of the ethical issues impeding the wider acceptance and adoption of AI HC. These concerns include data privacy, ownership, mishandling and breaches; AI users fear how their data will be accessed, shared or used; this has led to mistrust in adopting AI (Ermakova *et al*., 2013). Our respondents stressed the importance of protecting patients' data and obtaining their consent before accessing, using, or sharing these data, to ensure transparency and ethical implementation of AI in HC. To address this, the European Union has introduced the General Data Protection Regulation to protect patients' data and information across Europe (European Parliament and Council of the European Union, 2017); in the same direction, the US Department of Health and Human Services uses the Health Information Portability and Accountability Act to protect patients' data and information in the United States (Department of Health and Human Services, 2020). The results from this study have also revealed that bias in algorithms has been impeding the adoption of AI in HC. Bias occurs when an AI algorithm gives wrong results or discriminates against certain individuals or groups due to their gender, origin and background, and as a result, causes them harm (Cirillo *et al*., 2020). And this bias can be human-induced, during the development of an AI algorithm, or data-induced, due to its insufficiency (Mooney and Pejaver, 2018; Senders *et al*., 2018).

Our respondents stressed the importance of mitigating and eliminating this bias to ensure equitable access to quality care for all. These conformed to the direction given by the FDA to monitor AI models’ performance, assess their risks and retrain them (FDA, 2021a). As a result, interventions were introduced to evaluate and improve ML algorithms (FDA, 2021b). The EC has also published a set of proposed rules that include defining and classifying AI risks to govern and harmonize its application to address safety and human rights concerns (European Commission, 2021). This study has also revealed liability as one of the factors hindering the acceptance and implementation of AI in HC. These issues concern accountability and responsibility attribution, specifically when AI causes harm or undesirable effects to patients. The current literature has not clearly defined who should be held liable if AI causes harm to patients (Coeckelbergh, 2020; Pellegrino, 2004; Sparrow, 2007; Ting *et al*., 2017). Our respondents have therefore stressed the importance of addressing liability concerns to ensure transparency, accountability and trust for wider acceptance and implementation of AI in HC.

Lastly, this study recommends the modification of current AI legislation by enacting new laws and regulations as well as ensuring compliance with these regulations. AI should be continuously monitored and assessed to ensure its successful and ethical implementation in HC (Kaminski and Malgieri, 2021; Reisman *et al*., 2018; Lekadir, 2022). A fail-safe model needs to be built during the development processes of AI and ensure that all the necessary security and safety measures are considered before and after its deployment. This could be done through mandating periodic risk assessments, pre-market evaluations and post-market follow-ups (FDA, 2022). Our study also recommends subjecting AI users to continuous training and education to understand the benefits, risks and limitations of AI (FDA, 2022). The education and training programs should include knowledge of AI structure, input data requirements and interpretation of its results (Shi *et al*., 2020). HC professionals should be up to date with the latest developments in AI and understand how to interpret and use its results. This study also encourages investment in education and training initiatives for quality, ethical and responsible AI implementation in HC. The study has also cautioned against over-reliance on AI by encouraging human expertise integration in all stages and levels of AI design, development and implementation. This will promote the development of AI algorithms based on cocreation (Leone *et al*., 2021). And improving its credibility in terms of performance, generalizability and acceptance by users (Luo *et al*., 2021; Sun *et al*., 2022). The study also recommends defining limitations for AI, and humans should be responsible for making the final decisions regarding the AI results.

## Implications, limitations and future research directions

### Theoretical implications

This study has offered important theoretical implications by examining and linking AI and BM research areas. Although previous studies have explored the effects of digitization on firms' BM design (Laudien *et al*., 2024)**,** patient inclusion (Rachinger *et al*., 2019), BM innovation (Kulkov, 2023), but few have investigated its effects on HC outcomes and performance (Akinwale, 2020; Von Wedel *et al*., 2022). Consequently, this study has filled this gap by exploring the interconnections between AI and HC BMs through the lens of RBV, thereby improving our understanding of AI in HC and how these relationships are influencing the HC performance and outcomes. In doing so, this study has extended the debate on AI and how it can be used as an important asset to improve HC BMs; it has also extended the debate on HC AI ethical issues, and its implementation strategies, therefore, contributed to the theory in this specialization.

Secondly, contrary to most of the previous research that focused on one specific issue or a particular country, such as the UK’s HC professionals’ opinions on technological paradoxes and AI implementation (Singh *et al*., 2024), technology innovation and HC performance in Saudi Arabia (Akinwale, 2020), digitization effects on clinical and patient outcomes in Germany (Von Wedel *et al*., 2022) and BM innovation in India (Angeli and Jaiswal, 2016), this study sought and analyzed the opinions of HC professionals on the effects of AI on HC BM transformation and outcomes from different HC organizations across Europe and America using a RBV, thereby contributing to the theory through the incorporation of different and new perspectives across boundaries as well as expatiating on how the adoption of AI in HC is improving the quality of care, automation, efficiency, innovation and cost savings for both patients and HC organizations; in turn, improving our understanding of HC BM and how values are created, delivered and captured in this industry.

Thirdly, this study has discussed the ethical considerations for AI adoption in HC. These include data issues, biases in algorithms and liability concerns. This study has not only outlined and explained these challenges but has also offered recommendations on how they can be addressed by promoting and implementing educational and training programs for AI users, integrating human expertise, enacting laws, regulations and conducting periodic risk assessments, as well as ensuring compliance with laws and regulations, to promote trust in AI services, therefore contributed to the literature by extending the debate on AI ethical issues and its implementation strategies in HC.

## Practical implications

Exploring the interconnections between AI and HC BMs through the opinions of HC professionals using a RBV theory has offered important practical implications, serving as guidelines to HC policy and decision-makers for effective and efficient implementation of AI in their organizations. The findings have extensively and practically demonstrated how AI adoption in HC is increasing accessibility to HC, improving quality, patients' outcomes and experience, increasing efficiency and reducing costs. This provides enough evidence to support how AI capabilities can be adopted in HC to transform the creation, delivery and capturing of value. These findings will undoubtedly attract and influence HC organizations, HC professionals and patients to accept and adopt AI technologies. Therefore, this study has contributed to the practical application of AI in HC.

Moreover, by identifying and explaining ethical issues and their causes that constitute challenges, fear and constrain the acceptance and adoption of AI in HC, this study has advanced the study of the factors affecting the wider implementation of AI in HC. The results revealed that data privacy, bias in algorithms and liability issues are negatively impacting the adoption of AI in HC; these results will allow and help HC policy and decision-makers to have a comprehensive understanding of why HC professionals and patients are hesitant to adopt AI in their respective departments, work and needs. These discoveries will help them to address these issues by providing mechanisms to ensure security and protection of patients' data, mitigate and/or eliminate bias, as well as ensure accountability, to boost the confidence of HC AI users to promote its acceptance and adoption in HC organizations.

Lastly, this study has presented important guiding principles and strategies for effective and efficient implementation of AI in HC. This study recommends that HC policy and decision-makers should ensure that AI is continuously and periodically assessed for risks, efficiency and effectiveness. They should ensure that AI users’ interests are protected, their data privacy and security are ensured and their consents are obtained before accessing, sharing and/or using their data. They should also ensure accountability and transparency, adhere to the current laws and enact new regulations to manage and govern AI in HC. They should also implement educational and training programs for AI users. They should integrate human expertise and involve various stakeholders in the HC AI development and implementation. With these strategies and recommendations, this study provides important guidelines on how to successfully and ethically adopt and implement AI in HC, contributing immensely to the practice in this field.

## Limitations and future research

This study discussed how AI is transforming HC BMs and impacting outcomes, its ethical considerations and how it can successfully be implemented, contributing to the theory and practice. Despite these, this study is not without limitations; firstly, our study used the opinions of only 29 HC professionals. Despite their diversity, the sample is still small; therefore, future studies may consider employing a large sample that includes the opinions and perspectives of many HC professionals. Secondly, our study focused on advanced economies; its findings can therefore not be considered the same as those in the emerging economies; studies with a particular focus on these countries are therefore of paramount importance to compare the findings for a better understanding and comprehension of the impacts of AI on HC in these countries. Future research may therefore consider exploring these economies.

Thirdly, our study used a qualitative method based on data collected through open-ended essay questions. Future studies may consider employing a quantitative approach for comparisons and further understanding of the subject matter. Fourthly, our study focused on the opinions of HC professionals; the results will be corroborated and clear if the opinions of patients can be sought; we therefore urge future studies to investigate the patients’ perspectives on the impacts of AI in HC BMs and outcomes. Lastly, ethical considerations constitute serious challenges to the wider acceptance and adoption of AI in HC; more research is required in this direction. Future studies should therefore investigate issues, such as data security and privacy, liability and bias, and examine how they can be effectively and efficiently addressed to boost confidence and trust, thereby fostering greater adoption and implementation of AI in this industry.
